# Total reversal of internal carotid blood flow in a patient with severe stenosis of the brachiocephalic trunk

**DOI:** 10.1590/1677-5449.190124

**Published:** 2020-04-03

**Authors:** Carlos Eduardo Del Valle, Luiz Fernando Tosi Ferreira, Pedro Henrique Bragato, Sara Lucy de Oliveira, Fernanda de Oliveira Mauro, Walter Junior Boim de Araújo

**Affiliations:** 1 Universidade Federal do Paraná – UFPR, Hospital de Clínicas, Unidade de Ecodoppler Vascular, Curitiba, PR, Brasil.; 2 Universidade Federal do Paraná – UFPR, Hospital de Clínicas, Departamento de Cirurgia, Curitiba, PR, Brasil.

**Keywords:** brachiocephalic trunk, ultrasonography, Doppler, duplex, brain ischemia

## Abstract

Occlusions and severe stenoses of the innominate artery (brachiocephalic trunk) are rare and present with a wide variety of clinical manifestations, with hemispheric, vertebrobasilar and right upper limb ischemic symptoms. The most common cause is atherosclerosis. Duplex scanning may show right vertebral artery flow reversal, diminished subclavian flow, and several patterns of right carotid flow disturbance, including slow flow, partial flow reversal during the cardiac cycle and even complete reversal of flow in the internal carotid artery, which is a very uncommon finding. Herein, the authors describe the case of a female patient who was a heavy smoker, had severe stenosis of the brachiocephalic trunk, and had episodes of collapse. Besides the subclavian steal and partial flow reversal in the common carotid artery, duplex scanning also showed high-velocity reversed flow in the internal carotid artery during the entire cardiac cycle, a finding that is not reported in the literature at this magnitude.

## INTRODUCTION

Stenoses or occlusions of the brachiocephalic trunk (BCT, innominate artery) are rare and can present with a variety of clinical signs.^1^^-^3 Since the right subclavian artery and the right common carotid artery originate from the BCT, there may be manifestations of right upper limb ischemia, vertebrobasilar ischemia due to subclavian steal, or hemispheric symptoms related to carotid flow.^2^^,^^4^ Supplementary findings seen on vascular Doppler ultrasonography (USD) are highly variable. Flow reversal in the ipsilateral vertebral artery (subclavian steal phenomenon) may be accompanied by a phenomenon known as double steal, when perfusion of the ipsilateral common carotid artery also becomes dependent on the ipsilateral vertebral artery (in this case, the term “double steal” indicates that the vertebral artery perfuses both the upper limb and the right carotid).^5^^-^7 The changes detected by USD in the right carotid system can involve a variety of different abnormal flow patterns, including hypoflow with or without partial reversal of flow through the right common carotid artery, hypoflow or flow reversal through the right external carotid, and even cases in which the right internal carotid exhibits partial^8^^-^10 or total^4^^,^^11^ flow reversal.

This report describes a case of high-velocity reversed flow through the right internal carotid artery in a patient with asymmetric pulses and blood pressures in the upper limbs.

## CASE DESCRIPTION

The patient was a 58-year-old, hypertensive female smoker (20 cigarettes/day), with symptoms of frequent episodes of collapse. During outpatients follow-up at a cardiology service, it was observed that the patient had significant differences in upper limb pulses and blood pressure levels. The patient stated that she had no previous history of stroke or transitory ischemic events or any symptoms in the right upper limb.

**Figure 1:**
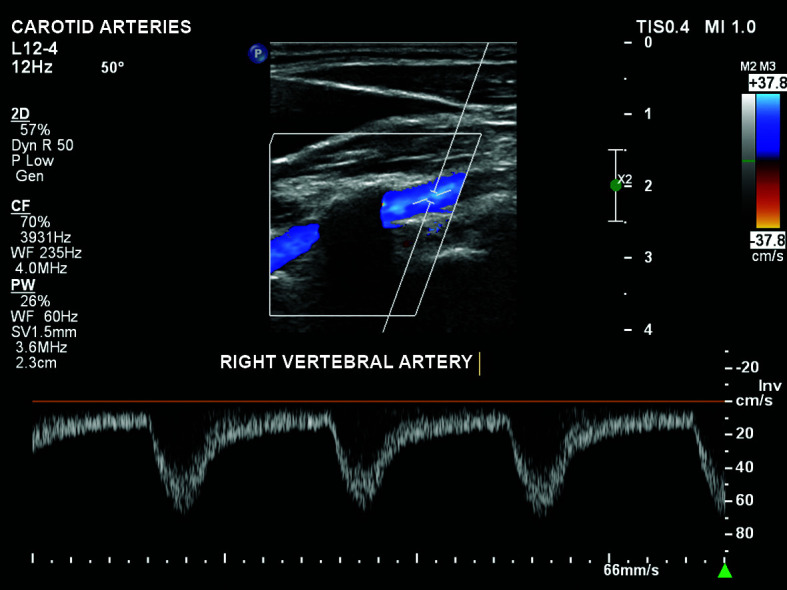
Reversal of flow in the right vertebral artery, constituting subclavian steal.
- partial flow reversal in the right common carotid, with caudal diastolic flow (Figure 2);

The difference in blood pressure levels in the upper limbs was investigated with USD of the carotid and vertebral arteries and the arteries of the upper limbs. The findings were as follows:

- complete reversal of flow in the right vertebral artery (subclavian steal phenomenon; Figure 1);

**Figure 2:**
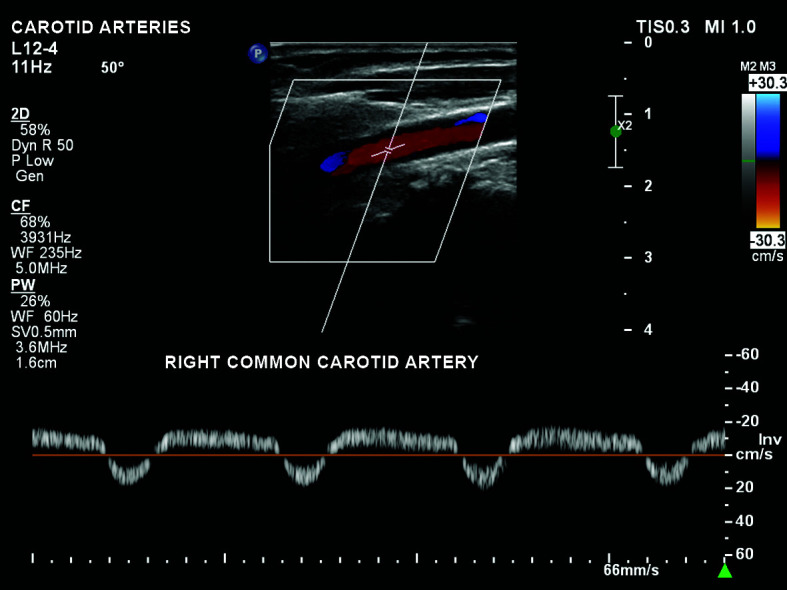
Partial reversal of flow in the right common carotid artery, with a to-and-fro appearance and anterograde flow during diastole only.
- complete reversal of flow in the right internal carotid (Figure 3);

**Figure 3:**
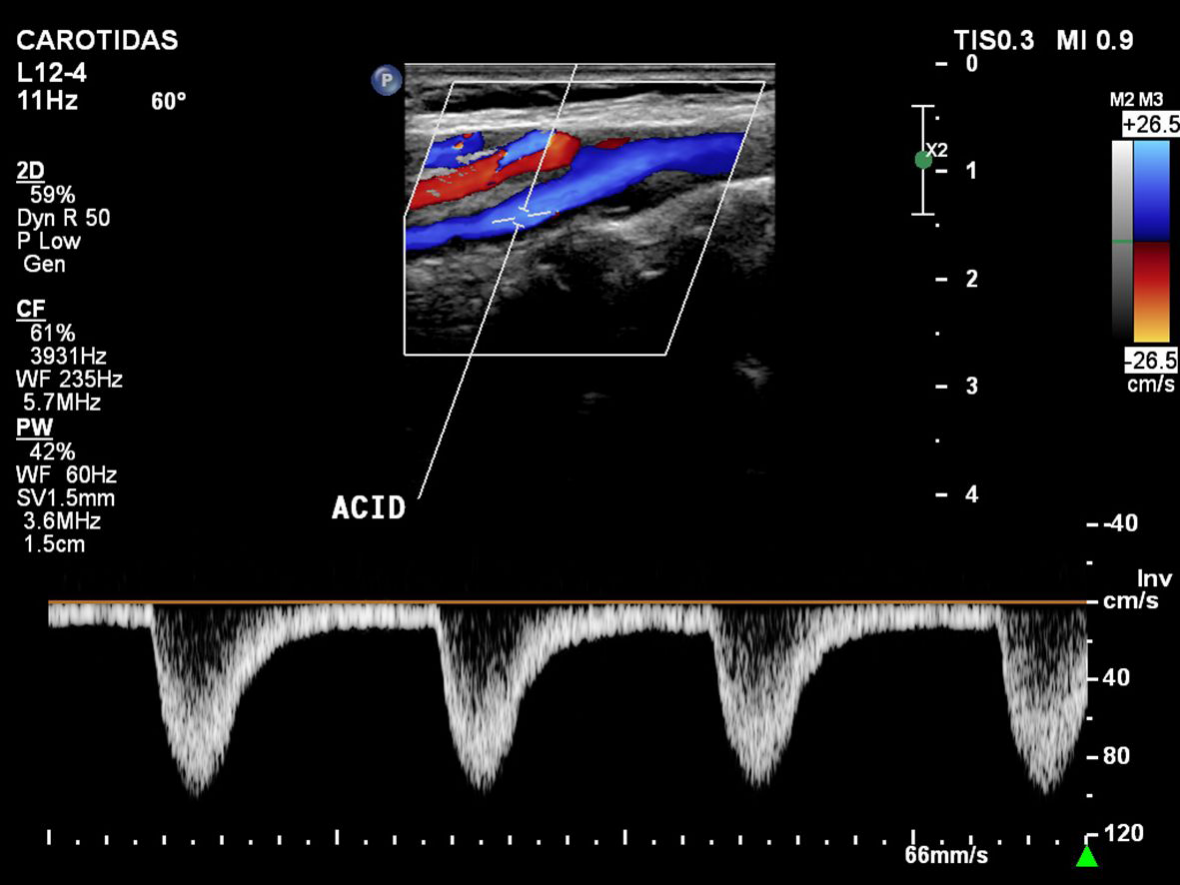
Total reversal of flow in the right internal carotid artery during the entire cardiac cycle, with systolic velocity close to 100 cm/s.
- low velocity and low resistance anterograde flow in the right external carotid (Figure 4);

**Figure 4:**
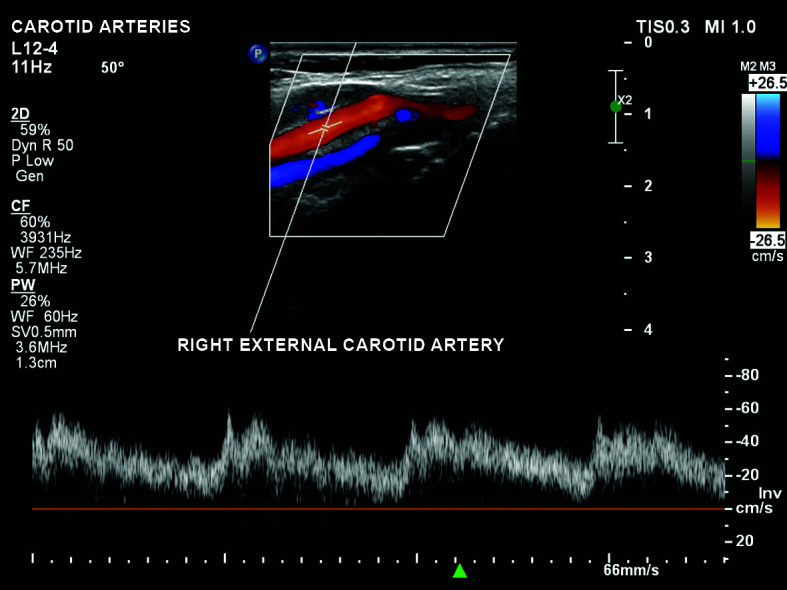
Low velocity anterograde flow in the right external carotid artery.
- low velocity hypoflow in the right subclavian (Figure 5);

**Figure 5:**
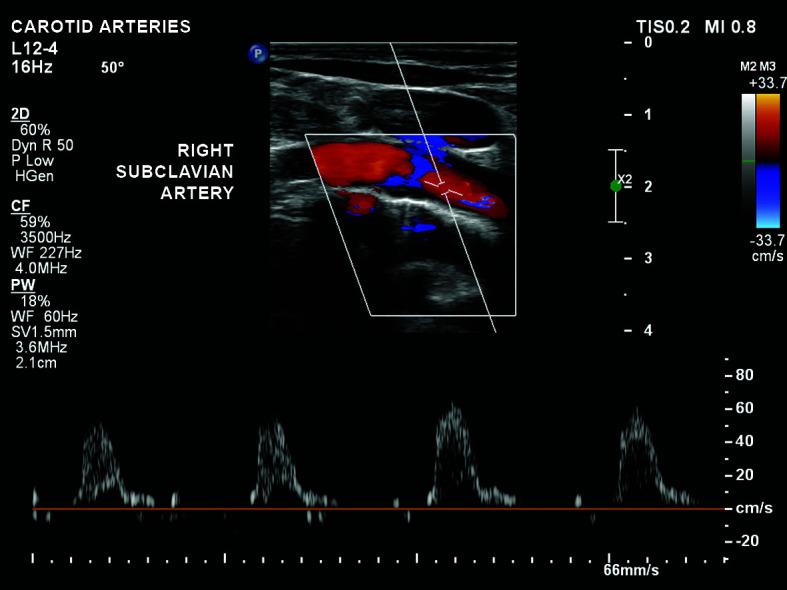
Low velocity hypoflow in the right subclavian artery.
- absence of flow in the BCT detectable by the method (Figure 6).

**Figure 6:**
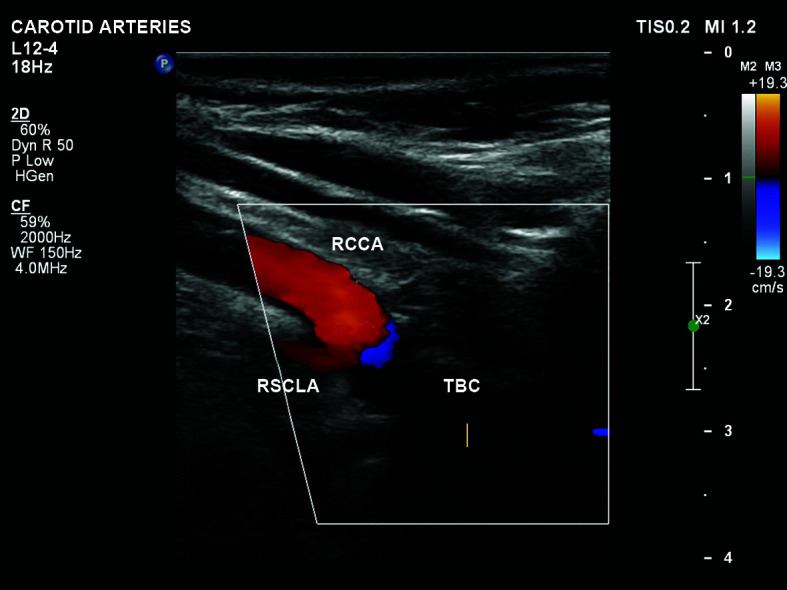
No flow in the BCT detectable by the method. RCCA = right common carotid artery; RSCLA = right subclavian artery; TBC = brachiocephalic trunk.

**Figure 7:**
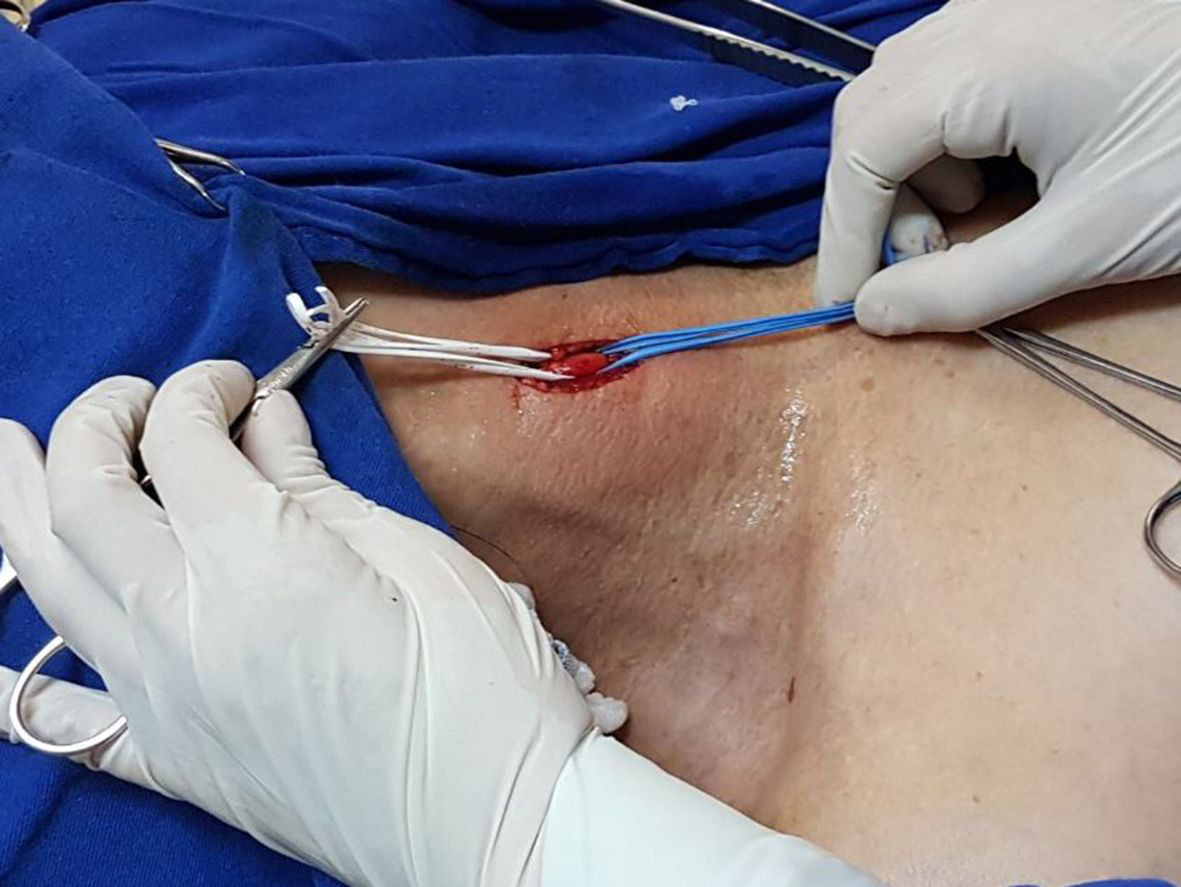
Hybrid treatment with cervical surgical access via the right common carotid artery.

**Figure 8:**
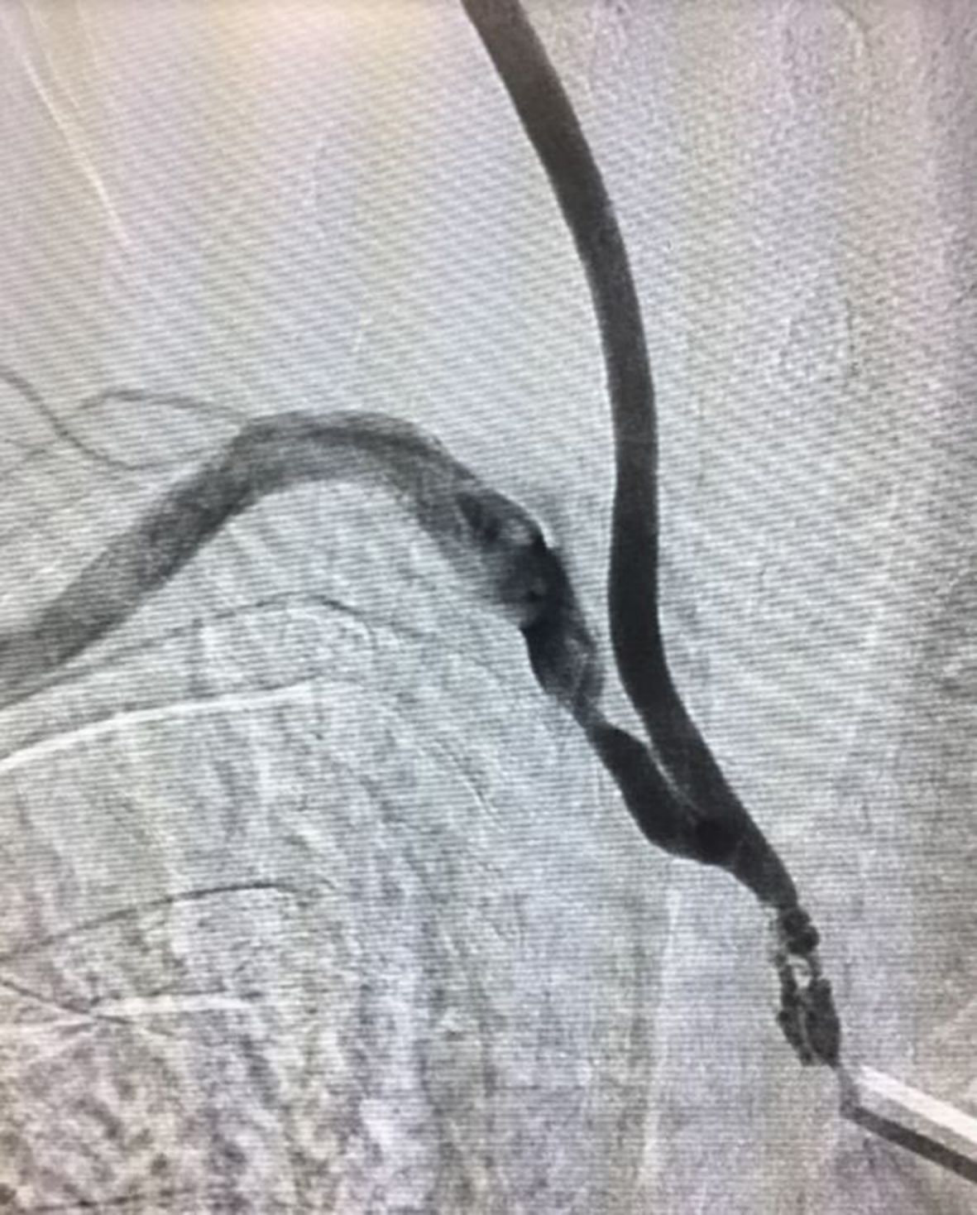
Initial arteriography confirming severe subocclusive stenosis of the brachiocephalic trunk and significant stenosis of the right subclavian artery.

**Figure 9:**
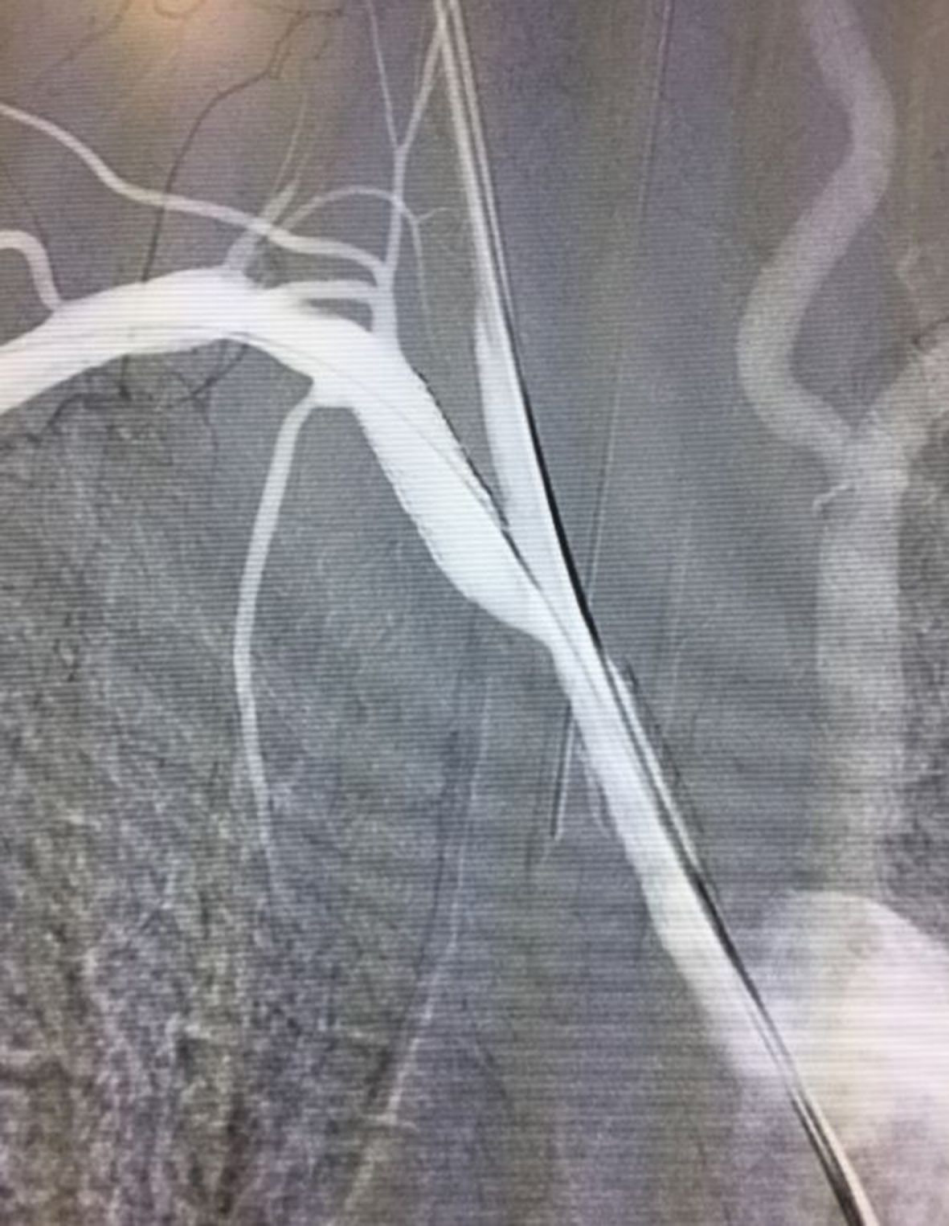
Final control arteriography demonstrating lesions corrected, by placement of a 6x25 mm Viabahn Gore covered stent in the brachiocephalic trunk and angioplasty of the right subclavian artery stenosis with a 7x17 mm Express LD balloon-expandable stent.

**Figure 10:**
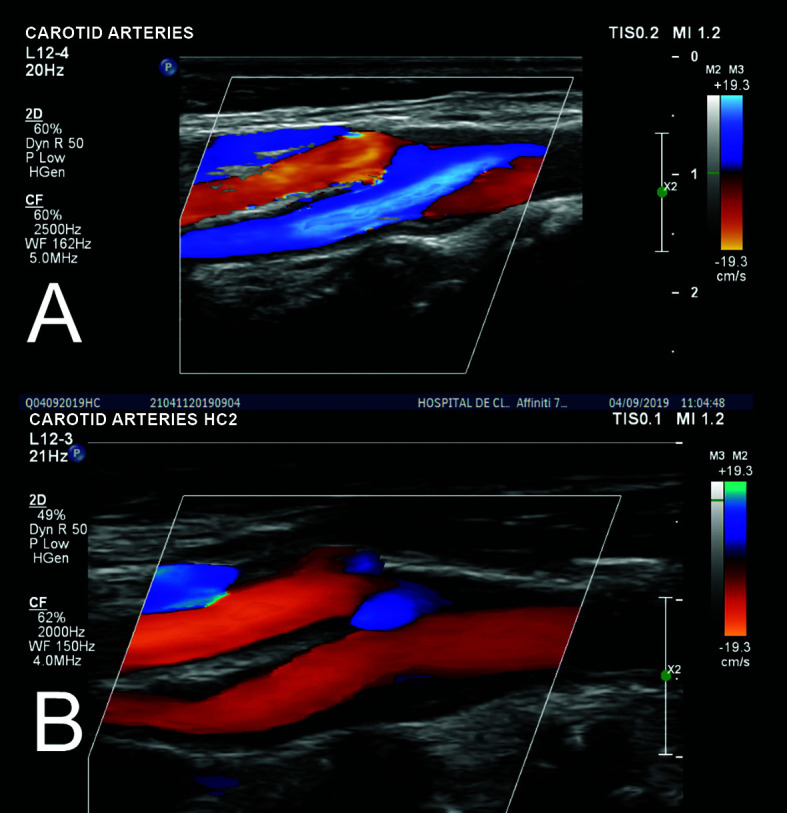
Comparison of the initial (A) and postoperative control (B) Doppler ultrasonography.

The investigation was continued using angiotomography, which showed atheromatous plaques with irregular surfaces and areas of ulceration causing severe stenosis in the BCT and at the origin of the right subclavian artery. The patient underwent hybrid endovascular treatment, with access obtained by dissection of the right carotid (Figure 7) and right brachial arteries, with confirmation of the lesions on the initial arteriography (Figure 8). Stenoses were treated by placement of a 6x25 mm Viabahn Gore covered stent in the BCT (because of the instability of the atheromatous plaques) and angioplasty with a 7x17 mm Express LD balloon-expandable stent in the right subclavian artery stenosis (Figure 9). The patient suffered no intercurrent conditions and postoperative control USD showed normalization of the flows through the right carotid artery (Figure 10). The patient has been in postoperative follow-up for 1 year and 10 months and reports that she has not had any further episodes of collapse.

## DISCUSSION

Severe stenoses and occlusions of the BCT are rare conditions and their true prevalence may well be unknown.^1^ In a study analyzing 30,000 USD examinations, the prevalence of obstructive disease involving the BCT was lower than 0.1%,^12^ while angiography studies suggest that they account for around 2.5% of lesions involving the extracranial circulation.^12^ The most common cause is atherosclerosis, while other possible etiologies include Takayasu’s Arteritis, giant-cell arteritis, radiotherapy-induced actinic fibrosis, and fibromuscular dysplasia.^4^

The most common symptoms include ischemia of the right upper limb, vertebrobasilar ischemia, and hemispheric symptoms in the territory corresponding to the right carotid system.^13^ In the case of the patient described here, the manifestations that prompted ordering of the USD examination were merely reduced pulses in the right upper limb and asymmetric blood pressures across the two upper limbs. The episodes of collapse were not initially attributed to presence of cerebrovascular disease, but as additional factors were revealed, this relationship was found to be present.

The most common USD finding in lesions involving the BCT is flow reversal in the right vertebral artery (subclavian steal phenomenon)^14^; but, in contrast with “single” subclavian steal, which occurs in obstructive lesions of the subclavian artery, there are also changes to flow in the right carotid system.^1^^,^^15^^,^^16^ Manifestations can range from reduction of peak systolic velocity in the carotid artery, with flow remaining anterograde, to cases of total reversal of flow, as reported in the present case. A hypothesis of a significant obstructive lesion of the BCT should always be considered in cases in which there is diffuse reduction of flow in the right carotid artery.^11^ If direct images of the BCT are difficult to obtain with a linear transducer, a convex or sector transducer can be used to try to directly document the lesion.

What makes this case particularly out of the ordinary, beyond the aforementioned rarity of this type of lesion, is the high velocity of the reversed flow in the right internal carotid artery (Figure 3). In our review of the literature, we found 24 articles that specifically mention changes found on vascular USD of obstructive lesions of the BCT (Table 1).^1^^-^24 Six of these describe hypoflow through the internal carotid artery without flow reversal in any phase of the cardiac cycle,^12^^,^^13^^,^^15^^,^^19^^,^^22^^,^^23^ and three only referred to the common carotid, without describing findings specific to the internal carotid.^1^^,^^14^^,^^17^ The most often reported finding (in 13 articles) was partial reversal of flow in the internal carotid artery, with retrograde flow during systole, but antegrade flow in diastole.^2^^,^^3^^,^^5^^-^10^,^^16^^,^^18^^,^^20^^,^^21^^,^^24^ Just two studies described complete reversal of flow in the internal carotid throughout the entire cardiac cycle: Grant et al.^11^ observed reversal with minimal diastolic flow and Borne et al.^4^ observed reverse flow throughout the whole cardiac cycle, but with systolic velocity of 37 cm/s. In our review, we did not find any cases of such high-velocity reversal as in the case described here, with systolic velocities approaching 100 cm/s (Figure 3).

**Table 1:** Results of a bibliographic review of Doppler ultrasonography findings for the internal carotid in patients with obstructive lesions involving the brachiocephalic trunk. Authors cited in alphabetic order.

**Authors**	**Year**	**Flow through the internal carotid artery**
Ackerstaff et al.^17^	1984	Only mentions the common carotid
Borne et al.^4^	2015	Total reversal
Brunhölzl and von Reutern^12^	1989	Hypoflow without reversal
Calin et al.^18^	2018	Partial reversal
Deurdulian et al.^2^	2016	Partial reversal
Esen et al.^5^	2016	Partial reversal
Filis et al.^6^	2008	Partial reversal
Grant et al.^11^	2006	Total reversal
Grosveld et al.^14^	1988	Only mentions the common carotid
Guedes et al.^1^	2016	Only mentions the common carotid
Han et al.^7^	2017	Partial reversal
Horrow et al.^19^	2008	Hypoflow without reversal
Maier et al.^20^	2014	Partial reversal
Racy^10^	2019	Partial reversal
Rawal et al.^13^	2019	Hypoflow without reversal
Rodriguez^3^	2016	Partial reversal
Schwend et al.^21^	1995	Partial reversal
Scoutt^15^	2019	Hypoflow without reversal
Sidhu e Morarji^22^	1995	Hypoflow without reversal
Tenny and Fleischmann^9^	2017	Partial reversal
Verlato et al.^23^	1993	Hypoflow without reversal
Uzun et al.^8^	2008	Partial reversal
Willoughby et al.^16^	2014	Partial reversal
Zwiebel and Pellerito^24^	2005	Partial reversal

No flow through the BCT detectable by the method was seen on USD; however, both angiotomography and arteriography via catheter demonstrated severe subocclusive stenosis, which constitutes pseudo-occlusion (an absence of flow on Doppler, but with patency demonstrated on angiography via catheter or on angiotomography, which is a phenomenon that occurs in very accentuated stenosis). The likelihood of pseudo-occlusion is possibly higher in the BCT than in the internal carotid artery, taking into account the vessel’s deep location.^3^

With regard to treatment, it is well-known that the BCT is a complex region to approach, because of its large diameter, short length, and anatomy including bifurcation to the subclavian and common carotid arteries.^25^ Another point that merits attention is transfemoral access, which may not be possible because of poor conditions along the route (femoral and iliac arteries and the aorta).^25^ In the present case, the decision to use a combined access, via the right upper limb and the right common carotid artery, was taken because of the ostial position of the lesion in the BCT in angiotomography, which is normally predictive of difficult catheterization via the femoral route, and also because this technique offers good protection against perioperative embolism. Thus, direct access to the vessels of the BCT via the right common carotid artery is an attractive option. The hybrid technique is safe and effective, offering protection against distal embolization via direct control of the common carotid artery with clamping and unclamping in a selective sequence.^25^ The patient had attributed her frequent episodes of collapse to presumed variations in blood pressure, but her symptoms disappeared after repair of the BCT stenosis and its repercussions for cerebrovascular hemodynamics, suggesting that the symptoms were caused by encephalic ischemia.
