# Supplementary material for: Total reversal of internal carotid blood flow in a patient with severe stenosis of the brachiocephalic trunk
Source: J Vasc Bras. 2020 Apr 3;19:e20190124. doi: 10.1590/1677-5449.190124 (PMC8202210; doi:10.1590/1677-5449.190124)
Supplement: Translation

# Translation: Inversão total do fluxo em artéria carótida interna direita em paciente com estenose grave do tronco braquiocefálico

**Authors:** Carlos Eduardo Del Valle, Luiz Fernando Tosi Ferreira, Pedro Henrique Bragato, Sara Lucy de Oliveira, Fernanda de Oliveira Mauro, Walter Junior Boim de Araújo

**Affiliations:** 1 Universidade Federal do Paraná – UFPR, Hospital de Clínicas, Unidade de Ecodoppler Vascular, Curitiba, PR, Brasil.; 2 Universidade Federal do Paraná – UFPR, Hospital de Clínicas, Departamento de Cirurgia, Curitiba, PR, Brasil.

**Keywords:** tronco braquiocefálico, ultrassonografia Doppler, isquemia encefálica

## Abstract

As estenoses graves e oclusões do tronco braquiocefálico (artéria inominada) são raras, e apresentam uma grande variedade de manifestações clínicas, com alterações relacionadas a isquemia cerebral hemisférica, vertebrobasilar e de membro superior direito. A causa mais comum é a aterosclerose. A ultrassonografia vascular com Doppler pode revelar inversão de fluxo na artéria vertebral direita, hipofluxo na subclávia, e vários tipos de alterações no fluxo da carótida direita, incluindo hipofluxo, inversão parcial do fluxo durante o ciclo cardíaco, e até mesmo inversão completa do fluxo na carótida interna, achado este bastante raro. Os autores descrevem o caso de paciente do sexo feminino, tabagista, com estenose grave do tronco braquiocefálico e crises de lipotimia. Além do roubo de artéria subclávia e do fluxo parcialmente invertido na carótida comum direita, a paciente apresentava exuberante fluxo invertido na carótida interna durante todo o ciclo cardíaco, achado este não encontrado na literatura em tamanha magnitude.

## INTRODUÇÃO

As estenoses ou oclusões de tronco braquiocefálico (TBC, artéria inominada) são entidades raras e podem apresentar sinais clínicos variados^1^^-^3. Pelo fato de o TBC originar a artéria subclávia direita e a artéria carótida comum direita, pode haver manifestações de isquemia de membro superior direito, isquemia vertebrobasilar por roubo de subclávia, ou sintomas hemisféricos relacionados ao fluxo carotídeo^2^^,^^4^. Os achados complementares na ultrassonografia vascular com Doppler (USD) são bastante variados. A inversão de fluxo na artéria vertebral ipsilateral (fenômeno do roubo de subclávia) pode ser acompanhada do chamado duplo roubo, quando a artéria carótida comum ipsilateral também passa a ter sua perfusão dependente da vertebral ipsilateral (o termo “duplo roubo” nesse caso significando que a artéria vertebral perfunde o membro superior e também a carótida direita)^5^^-^7. As alterações detectadas pela USD no sistema carotídeo direito podem assumir variados padrões de alteração de fluxo, entre eles hipofluxo com ou sem inversão parcial em artéria carótida comum direita, hipofluxo ou inversão de fluxo na carótida externa direita, e inclusive casos em que até mesmo a carótida interna direita apresenta inversão parcial^8^^-^10 ou total^4^^,^^11^ de fluxo.

O presente relato descreve um caso de inversão exuberante do fluxo na artéria carótida interna direita em uma paciente com assimetria de pulsos e de níveis pressóricos em membros superiores.

## DESCRIÇÃO DO CASO

Paciente do sexo feminino, 58 anos, hipertensa e tabagista (20 cigarros/dia), com sintomas de crises frequentes de lipotimia. Observou-se diferença significativa de pulsos e medidas pressóricas em membros superiores durante acompanhamento ambulatorial com o serviço de cardiologia. A paciente negava acidente vascular encefálico ou ataque isquêmico transitório prévio, e negava sintomas no membro superior direito.

Para investigar a diferença pressórica nos membros superiores, foi solicitada USD de artérias carótidas, vertebrais e arterial de membros superiores. Os achados foram:

- inversão completa de fluxo na artéria vertebral direita (fenômeno do roubo de subclávia; Figura 1);

**Figura 1:** Inversão do fluxo na artéria vertebral direita (ART. VERT. DIR), configurando roubo de subclávia.
- inversão parcial de fluxo na carótida comum direita, com fluxo diastólico caudal (Figura 2);

**Figura 2:** Inversão parcial do fluxo na artéria carótida comum direita (ACCD), com aspecto *to-and-fro* e fluxo anterógrado apenas durante a diástole.
- inversão completa de fluxo na carótida interna direita (Figura 3);

**Figura 3:** Inversão completa do fluxo na artéria carótida interna direita (ACID) durante todo o ciclo cardíaco, com velocidade sistólica próxima de 100 cm/s.
- fluxo anterógrado de baixa velocidade e baixa resistência na carótida externa direita (Figura 4);

**Figura 4:** Fluxo anterógrado de baixa velocidade da artéria carótida externa direita (ACED).
- hipofluxo de baixa velocidade na subclávia direita (Figura 5);

**Figura 5:** Hipofluxo de baixa velocidade na artéria subclávia direita (SUBCL DIR).
- ausência de fluxo ao método em TBC (Figura 6).

**Figura 6:** Ausência de fluxo detectável ao método no TBC. ACCD = artéria carótida comum direita; SUBCL D = artéria subclávia direita; TBC = tronco braquicefálico.

A investigação prosseguiu com angiotomografia, que evidenciou placa de ateroma com superfície irregular e áreas de ulceração gerando estenose grave em TBC e na origem da artéria subclávia direita. A paciente foi submetida a tratamento híbrido endovascular com acesso por dissecção em artéria carótida direita (Figura 7) e braquial direita, com a arteriografia inicial confirmando as lesões (Figura 8). As estenoses foram tratadas com implante de endoprótese revestida Viabahn Gore 6x25 mm em TBC (devido à instabilidade da placa de ateroma) e angioplastia com stent expansível por balão Express LD 7x17 mm em estenose da artéria subclávia direita (Figura 9). A paciente apresentou evolução sem intercorrências e foi submetida a USD de controle pós-operatório, que evidenciou normalização nos fluxos em artéria carótida direita (Figura 10). A paciente encontra-se com 1 ano e 10 meses de seguimento pós-operatório e relata não ter mais sofrido crises de lipotimia.

**Figura 7:** Tratamento híbrido com acesso cervical cirúrgico à artéria carótida comum direita.

**Figura 8:** Arteriografia inicial confirmando estenose grave suboclusiva do tronco braquiocefálico e estenose significativa na artéria subclávia direita.

**Figura 9:** Arteriografia de controle final demonstrando a correção das lesões, mediante implante de endoprótese revestida Viabahn Gore 6x25 mm em tronco braquiocefálico e angioplastia com stent expansível por balão Express LD 7x17 mm em estenose da artéria subclávia direita.

**Figura 10:** Comparação da ultrassonografia Doppler inicial (A) e controle pós-operatório (B).

## DISCUSSÃO

As estenoses graves e oclusões do TBC são lesões raras, e sua verdadeira prevalência pode ser desconhecida^1^. Em um estudo de 30.000 exames de USD, a prevalência de doença obstrutiva do TBC foi inferior a 0,1%^12^, com estudos de angiografia sugerindo que ela representa em torno de 2,5% das lesões da circulação extracraniana^12^. A causa mais comum é a aterosclerose, além de outras potenciais etiologias como arterite de Takayasu, arterite de células gigantes, fibrose actínica por radioterapia e displasia fibromuscular^4^.

Os sintomas mais comuns incluem isquemia de membro superior direito, isquemia vertebrobasilar e sintomas hemisféricos no território correspondente ao sistema carotídeo direito^13^. No caso da paciente descrita no presente trabalho, as manifestações que levaram à solicitação do exame de USD consistiam em apenas diminuição de pulsos no membro superior direito e assimetria da pressão arterial entre os dois membros superiores. De início, as crises de lipotimia não foram atribuídas à presença de doença cerebrovascular, mas com o decorrer dos fatos essa relação se revelou presente.

O achado mais comum na USD em lesões do TBC é a inversão de fluxo na artéria vertebral direita (fenômeno do roubo de subclávia)^14^; porém, ao contrário do roubo de subclávia “simples”, que ocorre em lesões obstrutivas da artéria subclávia, existem alterações de fluxo no sistema carotídeo direito^1^^,^^15^^,^^16^. Pode haver desde apenas redução na velocidade de pico sistólico na artéria carótida, com fluxo ainda anterógrado, até casos de inversão total do fluxo, como o relatado no presente caso. A hipótese de lesão obstrutiva significativa do TBC sempre deve ser considerada em casos nos quais o fluxo na artéria carótida direita é difusamente reduzido^11^. Quando imagens diretas do TBC são difíceis de obter com transdutor linear, pode-se usar transdutor convexo ou setorial, na tentativa de se obter documentação direta da lesão.

O que torna este caso particularmente distinto do habitual, afora a anteriormente mencionada raridade desse tipo de lesão, é a exuberância do fluxo invertido na artéria carótida interna direita (Figura 3). Em nossa revisão da literatura, foram encontrados 24 artigos que mencionam especificamente as alterações presentes na USD vascular em lesões obstrutivas do TBC (Tabela 1 1^-^24). Desses, seis descrevem hipofluxo na artéria carótida interna sem inversão do fluxo em nenhuma fase do ciclo cardíaco^12^^,^^13^^,^^15^^,^^19^^,^^22^^,^^23^, e três fazem referência apenas à carótida comum, sem descrever os achados especificamente na carótida interna^1^^,^^14^^,^^17^. O achado mais relatado (13 artigos) foi a inversão parcial do fluxo na artéria carótida interna, com fluxo retrógrado na sístole porém anterógrado na diástole^2^^,^^3^^,^^5^^-^10^,^^16^^,^^18^^,^^20^^,^^21^^,^^24^. Apenas dois trabalhos mostram uma inversão completa do fluxo na carótida interna durante todo o ciclo cardíaco: Grant et al.^11^ mostram uma inversão com mínimo fluxo diastólico, e Borne et al.^4^ mostram fluxo inverso durante todo o ciclo cardíaco, porém com velocidade sistólica de 37 cm/s. Em nossa revisão, não foram encontrados casos de inversão tão abundante quanto no caso presentemente descrito, com velocidades sistólicas se aproximando dos 100 cm/s (Figura 3).

**Tabela 1:** Revisão bibliográfica acerca dos achados da ultrassonografia Doppler na carótida interna em pacientes portadores de lesões obstrutivas do tronco braquiocefálico. Autores mencionados em ordem alfabética.

**Autor**	**Ano**	**Fluxo na artéria carótida interna**
Ackerstaff et al.^17^	1984	Menciona apenas carótida comum
Borne et al.^4^	2015	Inversão completa
Brunhölzl e von Reutern^12^	1989	Hipofluxo sem inversão
Calin et al.^18^	2018	Inversão parcial
Deurdulian et al.^2^	2016	Inversão parcial
Esen et al.^5^	2016	Inversão parcial
Filis et al.^6^	2008	Inversão parcial
Grant et al.^11^	2006	Inversão completa
Grosveld et al.^14^	1988	Menciona apenas carótida comum
Guedes et al.^1^	2016	Menciona apenas carótida comum
Han et al.^7^	2017	Inversão parcial
Horrow et al.^19^	2008	Hipofluxo sem inversão
Maier et al.^20^	2014	Inversão parcial
Racy^10^	2019	Inversão parcial
Rawal et al.^13^	2019	Hipofluxo sem inversão
Rodriguez^3^	2016	Inversão parcial
Schwend et al.^21^	1995	Inversão parcial
Scoutt^15^	2019	Hipofluxo sem inversão
Sidhu e Morarji^22^	1995	Hipofluxo sem inversão
Tenny e Fleischmann^9^	2017	Inversão parcial
Verlato et al.^23^	1993	Hipofluxo sem inversão
Uzun et al.^8^	2008	Inversão parcial
Willoughby et al.^16^	2014	Inversão parcial
Zwiebel e Pellerito^24^	2005	Inversão parcial

A USD não demonstrou fluxo detectável ao método no TBC; porém, tanto a angiotomografia como a arteriografia por cateter demonstraram estenose grave suboclusiva, o que configura pseudo-oclusão (ausência de fluxo ao Doppler porém com perviedade demonstrada em angiografia por cateter ou angiotomografia, fenômeno que ocorre em estenoses muito acentuadas). No caso do TBC, a chance de pseudo-oclusão é talvez maior do que na artéria carótida interna, tendo em vista a localização profunda daquele vaso^3^.

No que se refere ao tratamento, é sabido que o TBC é uma região de abordagem complexa, dado seu grande diâmetro, comprimento restrito e a anatomia com bifurcação para artérias subclávia e carótida comum^25^. Outro alvo de atenção é o acesso transfemoral, que pode ser impossibilitado devido a más condições no trajeto vascular (artérias femoral, ilíaca e aorta)^25^. No presente caso, a opção pelo acesso combinado pelo membro superior direito e pela artéria carótida comum direita foi considerada devido à natureza ostial da lesão do TBC na angiotomografia, o que normalmente prediz cateterização difícil pela via femoral, além de oferecer boa proteção contra embolismo transoperatório. Assim, o acesso direto aos vasos do TBC via artéria carótida comum direita se torna uma opção interessante. A técnica híbrida é segura e efetiva, oferecendo proteção contra embolização distal por meio do controle direto da artéria carótida comum com clampeamento e desclampeamento em sequência seletiva^25^. A paciente atribuía suas crises frequentes de lipotimia a uma suposta variação na pressão arterial, porém os sintomas desapareceram após a correção da estenose de TBC e de suas repercussões na hemodinâmica cerebrovascular, o que sugere que os sintomas eram decorrentes de isquemia encefálica.
